# Influenza vaccine in cardiovascular disease: Current evidence and practice in India

**DOI:** 10.1016/j.ihj.2024.11.247

**Published:** 2024-11-28

**Authors:** Ambuj Roy, Satyavir Yadav

**Keywords:** Influenza, Cardiovascular disease, Influenza vaccine, Heart failure, Coronary artery disease

## Abstract

Influenza is a common trigger for cardiovascular events. Temporal association studies of influenza and cardiovascular events have well documented this phenomenon. More recently, randomised clinical trials of influenza vaccine have shown the benefit of immunisation in reducing recurrent cardiovascular events, especially in patients with acute coronary syndrome. Despite this overwhelming benefit, its uptake in India is very low. This could be due to a lack of awareness and paucity of evidence of its benefit in tropical countries like India, where the influenza season is variable and spread throughout the year. In this review, we explore these aspects of influenza and cardiovascular diseases and discuss the way ahead.

## 1 Introduction

**Fig. 1:**
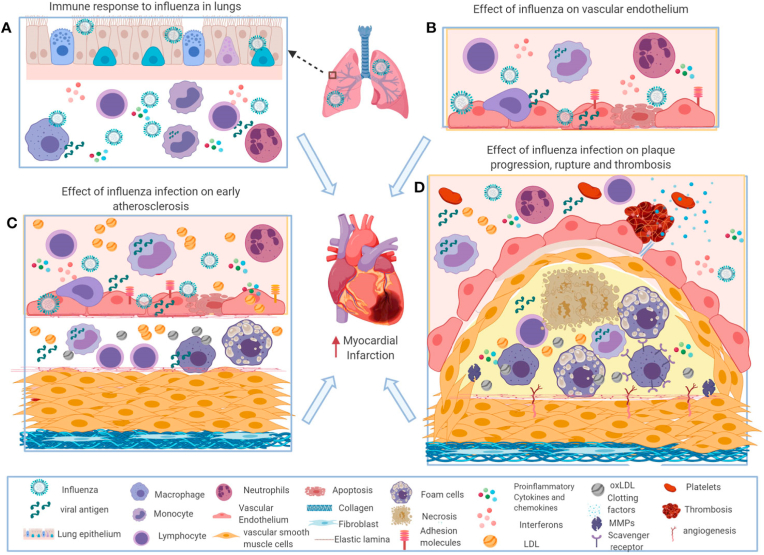
Potential immune mechanisms of influenza-induced exacerbation of atherosclerosis (With permission from Frontiers Publishers)^2^. Central figure: Influenza vaccine in cardiac conditions.

Cardiovascular disease (CVD) is the leading cause of death in India and globally, with coronary artery disease (CAD) being the most common CVD^1^. Multiple factors contribute to this burden and are called risk factors. While the major risk factors-tobacco use, hypertension, diabetes and dyslipidemia are the leading contributors to this burden, other risks also trigger atherosclerotic events that are not well characterised. One of the risks is infection, which may perpetuate or trigger acute CVD events through multiple mechanisms (Fig. 1).^2^ Influenza-associated mortality globally is estimated at 290,000 to 650,000, with the maximum mortality rate in sub-Saharan Africa and Southeast Asia.^3^ Annual influenza-associated respiratory and circulatory deaths are approximately 130,000 in India.^4^ This article discusses influenza's impact on CVD and influenza vaccination's impact on CVD prevention. We also discuss the Indian perspective of influenza particularly the variable seasonality compared to temperate countries and its impact on the vaccination across different parts of the country.

## 2 Influenza and cardiovascular disease: association and postulated mechanism

William Osler outlined the pivotal role of infection in the development of atherosclerosis^5^ more than a century ago. Epidemiological studies like the classic 35-city survey in the USA public health reports of 1932, which included nearly 2.5 crore inhabitants and weekly reports of all-cause deaths from 1917 to 1929, demonstrated an increased incidence of non-respiratory deaths after every influenza epidemic^6^ with some epidemics accruing as high as 40 % excess deaths to non-influenza causes. Several of these deaths were linked to organic heart disease, suggesting an increase in CVD mortality after each epidemic. Similar results were reported in more recent studies with community surveillance in Atherosclerosis Risk in Communities,^7^ demonstrating a 5 % absolute monthly increase in influenza activity led to a 24 % increase in heart failure hospitalisation. In a self-controlled case series, Kwang et al demonstrated that an episode of influenza increases the odds of having a myocardial infarction (MI) six times over the next seven days.^8^

Influenza has multiple postulated effects on the vascular system. Studies on animals have shown that inoculation with the influenza virus leads to an infiltration of macrophages and T cells, fibrin deposition, platelet aggregation, and thrombosis in atherosclerotic plaques, which is strikingly similar to what is observed in coronary plaques after a fatal MI.^9^^,^^10^ Influenza increases the expression of various chemokines, adhesion molecules ICAM1, VCAM-1, and E-selectin in human coronary endothelial cells. The body's immune system triggers the production of interferons and various inflammatory mediators to attract immune cells such as macrophages, neutrophils, and natural killer (NK) cells to the infection site to combat the virus. However, excessive influx of immune cells and uncontrolled production of inflammatory cytokines and chemokines increase the formation of foam cells, stimulate smooth muscle proliferation, contribute to plaque rupture, and increase the risk of thrombosis, potentially leading to acute myocardial infarction.^11^ Viral infections can also cause type 2 MI by increasing metabolic demand due to fever, tachycardia, and hypoxemia

These infections can also have adverse outcomes in the intermediate and long term. A large study from the US of approximately 6000 elderly patients with a follow-up of 11.9 years revealed a pronounced increase in new-onset heart failure in intermediate and long-term follow-up, irrespective of the severity of pneumonia and independent of traditional risk factors such as coronary artery disease.^12^

## 3 Impact of influenza vaccination among CAD and heart failure patients

Vaccination studies and their impact on cardiovascular outcomes further prove the association between influenza and CVD. Most earlier studies were observational; however, recent well-powered randomised trials have strengthened the association between influenza and CVD.

1) Influenza Vaccination trials in CAD patients.

The most recent and largest trial is the IAMI trial, which included 2571 patients with a recent myocardial infarction, were randomised to influenza vaccine or placebo saline injection within 72 h of symptom onset. The study showed a 28 % reduction in the primary outcome, a composite of all-cause death, heart attack, and stent thrombosis at 12-month follow-up, and a 42 % reduction in all-cause and cardiovascular mortality.^13^

A meta-analysis of RCTs of influenza vaccination in patients of CAD was done after the IAMI trial. It showed a 34 % % reduction (3.6 % vs 5.4 %; RR,0.66; 95%CI,0.53–0.83; *p* < 0.001) in major adverse cardiovascular event (MACE)in those who received the influenza vaccine. This 1.8 % absolute risk reduction means that 56 patients must be immunised to prevent 1 major cardiovascular event. There was a significant treatment interaction by the presence of ACS. In patients with ACS, the relative and absolute risk reduction with influenza vaccine were 45 % and 4.5 %, respectively, in MACE, suggesting the need to immunize only 23 patients of ACS to prevent one MACE. However, the patients without ACS did not show any MACE reduction in this meta-analysis. Further, the meta-analysis revealed that the patients receiving influenza vaccination had a cardiovascular mortality of 1.7 % versus 2.5 % in those not immunised which did not meet statistical significance (RR, 0.74; 95 % CI, 0.42–1.30; *p* = 0.29). However, in a subgroup analysis of patients with recent ACS, there was a 56 % reduction in cardiovascular mortality (2.6 % vaccine vs 5.4 % placebo/control; RR, 0.44; 95 % CI, 0.23–0.85; *p* = 0.01) with a number needed to treat of 36 to prevent 1 cardiovascular death.^14^

This benefit is better than any other established secondary prevention therapies such as aspirin, P2Y12 inhibitors, statins, beta-blockers, angiotensin-converting enzyme inhibitors, and angiotensin receptor blockers, all of which reduce mortality by 20–41 % in RCTs.^15^, ^16^, ^17^, ^18^, ^19^ This immense benefit should encourage wider use of the vaccine in these groups of patients. Also, other trials should test the reproducibility of these results in other atherosclerotic CVD settings like stable CAD, post-stroke and peripheral artery disease (PAD). Also, its reproducibility in non-temperate geographic locations must be tested where such trials have not been done. The various RCTs done are summarised in Table 1.

2) Influenza vaccination in Heart Failure patients

**Table 1:** Summary of RCTs of influenza vaccine in population with cardiovascular diseases.

Trial, Year	Sample size (Location)	Patients characteristics	Timing of recruitment	Study duration (months)	Effects/Results
FLUVACS, 2004^37^ Randomised, prospective, multicentre, parallel-group, and controlled pilot study	292 (Argentina)	ACS (66 %) and Elective PCI (34 %)	May–September (SH vaccine)	12	Significant reduction in cardiovascular death compared to controls (6 % vs 17 %, *p* = 0.002, hazard ratio 0.34, 95 % CI 0.17 to 0.71) More benefit with high TIMI score and NSTEMI (95%CI:0.13{0.03–0.52})
FLUCAD, 2008^38^ Randomised, double-masked, placebo-controlled study	658 (Poland)	CAD (ACS∼26 %; Stable CAD∼74 %)	October–February (NH Vaccine)	12	No significant reduction in cardiovascular death (3.0 vs. 5.8 %, *p* = 0.13) No significant reduction in MACE (cardiovascular death, acute myocardial infarction or coronary revascularization )(3.00 vs. 5.87 %, respectively); HR of 0.54 (95 % CI: 0.24–1.21; *P* = 0.13). Significant reduction in coronary ischemic events comprising of combination of MACE or hospitalisation for myocardial ischemia; 6.02 vs. 9.97 %, HR of 0.54 (95 % CI: 0.29–0.99, *P* = 0.047)
Phrommintikul et al, 2011^39^ Prospective randomised open with blinded endpoint study	439 (Thailand)	CAD (Recent ACS within past eight weeks)	November–October	12	Significant reduction in major cardiovascular events [unadjusted hazard ratio (HR) 0.70 (95 % CI: 0.57–0.86)]; however, no reduction in CV death
IVVE 2022^21^ Randomised, double-blind, placebo-controlled trial	5129 (China, India, Philippines, Saudi Arabia, United Arab Emirates,Kenya, Mozambique, Nigeria, Uganda, Zambia)	Symptomatic Heart Failure (NYHA II-IV)	October–December (NH vaccine) *AND* April–June (SH Vaccine)	36	No reduction in primary composite outcome of cardiovascular death, non-fatal myocardial infarction, and non-fatal stroke. (14.8 % vs ‵16 %; HR = 0·93 [95 % CI 0·81–1·07]; *p* = 0·30) Significant reduction in the composite outcome during the flu season (7·5 % vs 9·3 %; HR 0·82 [95 % CI 0·68–0·99]; *p* = 0·038).
IAMI, 2021(13) Randomised, double-blind, placebo-controlled trial	2532 (Sweden, Denmark, Norway, Latvia, United Kingdom, Czech Republic, Bangladesh, and Australia)	Recent MI(99.5 %) or high-risk stable CAD(0.5 %)	September–February & May–September in Australia, Bangladesh	12	Significant reduction in MACE [HR, 0.72 (95 % CI: 0.52–0.99)] Significant reduction in all-cause mortality [HR, 0.59 (95 % CI: 0.39–0.89)] and cardiovascular mortality [HR, 0.59 (95 % CI: 0.39–0.90)].

ACS- Acute Coronary Syndrome; PCI- Percutaneous Coronary Intervention; CAD- Coronary Artery Disease; MI- Myocardial Infarction; MACE **-**Major Adverse Cardiovascular Events; TIMI-The thrombolysis in myocardial infarction; NSTEMI-Non-ST-elevation myocardial infarction; FLUVACS-FLU Vaccination Acute Coronary Syndromes and Planned Percutaneous Coronary Interventions Study; FLUCAD-Influenza Vaccination in Prevention From Acute Coronary Events in Coronary Artery Disease Study; IVCAD-Efficacy of Influenza Vaccination in Reducing Cardiovascular Events in Patients With Coronary Artery Diseases; IVVE-Influenza Vaccine in Patients With Heart Failure to Reduce Adverse Vascular Events; IAMI-Influenza vaccination After Myocardial Infarction trial; RCT- Randomised Control Trial.

The influenza vaccine is considered the standard of care for heart failure patients. However, there is limited evidence to support the recommendation. Fukuta et al.,^20^ in a systematic review of observational studies, reported a 24 % reduction in all cause mortalityat one year and a 20 % reduction in long-term all-cause mortality. The reduction in mortality was higher in the flu season (48 %) compared to the non-flu season (21 %). There was a 16 % reduction in cardiovascular hospitalisation during the flu season. There was no large randomised trial in heart failure patients until the IVVE trial to determine the impact of the influenza vaccine on outcomes in tthem. It was also the first influenza vaccine trial done outside the temperate zone in locations without a well-defined single flu season. The participants received the flu vaccine for up to three seasons and were followed for cardiovascular death, non-fatal myocardial infarction, non-fatal stroke, and hospitalisation for heart failure. The trial included over 5000 patients with symptomatic heart failure from 30 sites in 10 countries. The trial did not show a reduction in the co-primary outcomes of a composite of cardiovascular death, non-fatal heart attack, or non-fatal stroke or heart failure hospitalisation at 36 months. However, when analysed by season, as often vaccine trials are, there was a significant 18 % reduction in the combined end point of cardiovascular death, non-fatal heart attack, and non-fatal stroke. There was also a significant decrease in all-cause and cardiovascular death and incidence of pneumonia.^21^ A considerable observation supportive of the vaccine's protective effect was that the vaccine's benefit was observed only in the season where the predominant influenza-causing strain matched the vaccine strain received and not when there was a mismatch of the vaccine strain and the circulating flu virus. This trial was carried out in countries in Africa, the Middle East, and India, where there is no clear seasonality of the flu season, which may account for the lower-than-expected response to the vaccine. Also, patients with heart failure may have compromised immune response to the vaccine, and high doses of the vaccine have been tried in trials in such patients as in the INVESTED trial.^22^

An updated meta-analysis of influenza vaccination to investigate the effectiveness of influenza vaccine in patients with cardiovascular disease, after the large IAMI and IVVE trials. included eight randomised control trials and 14,420 patients. The results of the meta-analysis revealed a 25 % reduction in major adverse cardiovascular events (MACE) [HR 0.75 (95 % CI 0.57–0.97)]. There was a numerically lower cardiovascular [HR 0.77 (0.39–1.50)] and all-cause mortality [HR 0.84 (0.54–1.33)] reported; however, it was not statistically significant. The results of the IVVE trial, which was the largest trial in the meta-analysis and contributed to one-third of patients, were not analysed by flu season and could have impacted the result.^23^

## 4 Current recommendations

The World Health Organization (WHO) recommends annual vaccinations for pregnant women, children aged 6 months to 5 years, people over 65 years, people with chronic medical conditions, and health workers.^24^ The Government of India's recommendation is to vaccinate high-risk priority groups like health care workers, pregnant women, and those with chronic diseases, including "heart disease" patients (Table 2).^25^ It also mentions that it is desirable to provide influenza vaccination to individuals ≥65 years of age and children aged six months to 8 years of age.

**Table 2:** List of the high-risk groups as per the Ministry of Health and Family Welfare, India eligible for influenza vaccine.

List of the high-risk influenza groups as per the Ministry of Health and Family Welfare, India
Healthcare workers working in hospitals/institutional settings (doctors, nurses, paramedics) with the likelihood of exposure to influenza virus. These include
All medical and paramedical personnel working in the emergency department of identified hospitals treating influenza cases
All medical and paramedical personnel working in ICU and isolation wards managing influenza patients
All personnel identified to work in screening centres that would be set up to categorise patients during seasonal influenza outbreaks.
Those treating/managing high-risk groups
Laboratory personnel working in virological laboratories testing suspected influenza samples
Rapid-response team members were identified to investigate outbreaks of influenza.
Drivers and staff of vehicles/ambulances involved in the transfer of influenza patients
Vaccination is recommended for Pregnant women, irrespective of the duration of pregnancy
People with chronic illnesses, such as chronic obstructive pulmonary disease, bronchial asthma, heart disease, liver disease, kidney disease, blood disorders, diabetes, and cancer, and those who are immunocompromised
In children with chronic diseases such as asthma; neurodevelopmental conditions such as cerebral palsy, epilepsy, stroke, mentally challenged, etc.; heart diseases such as coronary artery disease and congestive heart failure; blood disorders such as sickle cell disease; diabetes, metabolic disorders; all immunocompromised children; malignancy receiving immuno-suppressive therapy; kidney disorder; and liver disorder
Vaccination is desirable for: Elderly individuals (≥65 years of age)
Children aged 6 months to 8 years of age
ICU: Intensive care unit, CHD: Coronary heart disease, CHF: Congestive heart failure

## 5 Indian perspective

Despite the recommendations of regulatory authorities and professional societies, the uptake of the influenza vaccine is very low in India. The substudy of the PARADIAGM heart failure trial showed an abysmal 0.2 % use of influenza vaccine among heart failure patients in India. In comparison, the overall use in the trial was 21 %, with the highest use in European countries like the Netherlands (78 %) and Great Britain (77 %).^26^ Major heart failure and acute coronary syndrome registries from India, like the Indian College of Cardiology and National Heart Failure Registry and the Kerala ACS registry have not included the use of vaccination in their reports.^27^^,^^28^ It is essential to educate and disseminate knowledge about influenza vaccines among healthcare practitioners and vulnerable patients across India to ensure that the benefits of the vaccination are accrued in CVD patients.

### 5.1 Influenza seasonality and vaccination timing in India

India's geographic location and weather conditions make the influenza season heterogeneous with no one single peak as in temperate countries. There may be more than one influenza peak as also low level of transmission through the year, with influenza activity varying by location and onset of monsoon.^29^ This is important in context of appropriately timing the immunization in the population before the peak season. The immune response usually sets in within two weeks and lasts for six months; thus, the vaccination must preferably be administered as per the peak season of that geographic location. Two vaccines are marketed in India-one in April is the Southern Hemisphere (SH) vaccine, and the other in October is the Northern Hemisphere (NH) vaccine. The viral composition of these vaccines is based on the latest viruses circulating and infecting humans, and it is recommended by the World Health Organisation (WHO).

The northernmost part of India has a peak influenza season from December to April, and thus, people should be vaccinated with the NH influenza vaccine from October to December. This would include the Union Territory of Jammu and Kashmir and Ladakh, the states of Himachal Pradesh and Uttarakhand and parts of Punjab. Most other parts of India have an influenza season following the monsoon, which peaks between July and November. Thus, people living in these locations should receive the Southern Hemisphere (SH) vaccine from April onwards. Tamil Nadu, with rains due to the retreating north-east monsoon, has a peak influenza season from September to December, and the SH vaccine should be administered to them from May to August.^29^, ^30^, ^31^ The peak season varies in India, and some areas may have more than one peak, both during the post-monsoon and winter seasons.^32^^,^^33^

### 5.2 Vaccination and immunological response

The immunogenicity of influenza vaccine is affected by several factors, including vaccine factors and individual factors. Discussing the details of this is beyond the scope of this review. However, one must be aware of clinically relevant vaccine efficacy factors. There have been concerns about reduced immunogenicity among elderly heart patients and heart failure patients labelled as immunosenescence. Additionally, patients on steroids may also have a lower immunogenicity, as may happen in patients with idiopathic myocarditis or cardiac sarcoidosis. Another point of concern is the impact of statin, an immunomodulator. Some studies have raised concerns that statin use was associated with lower vaccine efficacy^34^; however, this is not borne out in other studies.^35^ A large randomised trial was conducted to address these concerns of vaccine efficacy in patients with recent acute myocardial infarction (AMI) or heart failure (HF) hospitalisation. One arm received a trivalent influenza vaccine with four times the dose of hemagglutinin antigen to improve immune response compared with the standard dose quadrivalent vaccine in the other arm, and major adverse cardiovascular outcomes were assessed. The two arms had no difference in vaccine efficacy.^22^ Thus, there are no recommendations for high-dose vaccine use in these patients.

The immunity post-influenza vaccine sets in by two to four weeks and wanes by over 50 % by six months post-vaccination.^36^ Thus, whether a single influenza vaccine suffices for high-risk cases and covers both post-monsoon and winter influenza seasons may be debated in countries like ours with a dispersed influenza activity and more than one peak season. However, due to a dearth of evidence, biannual vaccination has no evidence support, and recommendations are for a single annual immunisation.

### 5.3 Way forward for influenza vaccination in CVD in India

The evidence supporting the influenza vaccine is strong, especially post-acute coronary syndrome. Based on current evidence and guidelines, it is crucial to disseminate knowledge among physicians, patient help groups and policymakers to improve vaccine uptake in patients with cardiovascular diseases. This could be done through knowledge sharing and advocacy through national guidelines. Discharge checklists of patients with heart failure and CAD should include influenza vaccination. However, it is essential to note that most studies and evidence of influenza vaccine come from Western countries with a temperate climate and a fixed and predictable influenza season. The only available trial from countries out of the temperate zone is the IVVE trial in heart failure patients. This study, as discussed, was negative for the primary outcome studied, though there was a reduction in the primary outcome during the influenza season. This calls for locally generated evidence through RCTs done within the country to assess the influenza vaccine's utility in our population. This will help improve awareness and reduce hesitancy among physicians and the population regarding vaccine use, thus improving vaccine uptake, which is currently low in India. It will provide greater confidence to policymakers to formulate appropriate guidelines for its use. In this context, we are conducting a large multicentric randomised clinical trial across sites in India titled "Influenza vaccine to reduce cardiovascular events in patients with recent myocardial infarction: A multicentric randomised, double-blind, placebo-controlled trial (FLUENTI-MI)." The study will assess the effect of the pre-season influenza vaccine on major adverse cardiovascular events in patients with recent MI.

## Declaration of competing interest

The authors declare that they have no known competing financial interests or personal relationships that could have appeared to influence the work reported in this paper.
